# Physical Sins

**Published:** 1892-04

**Authors:** 


### PHYSICAL SINS.

Perhaps nothing will so much hasten the time when body and mind will both be adequately cared for, as a diffusion of the belief that the preservation of health is a duty. Few seem conscious that there is such a thing as physical morality. Men's habitual words and acts imply the idea that they are at liberty to treat their bodies as they please. Disorders entailed by disobedience to nature's dictates they regard simply as grievances, not as the effects of a conduct more or less flagitious. Though the evil consequences inflicted on their dependents, and on future generations, are often as great as those caused by crime, yet they do not think themselves in any degree criminal. It is true that in the case of drunkenness, the viciousness of a bodily transgression is recognized; but none appear to infer that if this bodily transgression is vicious, so, too, is every bodily transgression. The fact is, that all breaches of the laws of health are physical sins. When this is generally seen, then and perhaps not till then, will the physical training of the young receive deserved attention.—*Herbert Spencer.*

